# Impact of ERCP simulator training on early ERCP learning curves of novice trainees: a cohort study

**DOI:** 10.1055/a-2114-2842

**Published:** 2023-08-01

**Authors:** Sophia Elisabeth van der Wiel, Erik Rauws, Stijn Van Gool, Dong Wang, Bing Hu, Leena Kylanpaa, George J.M. Webster, Martin James, Arjun Dave Koch, Marco Bruno

**Affiliations:** 1 6993 Gastroenterology and Hepatology, Erasmus Medical Center, Rotterdam, Netherlands; 2 26066 Gastroenterology and Hepatology, Amsterdam UMC Locatie AMC, Amsterdam, Netherlands; 3 70515 Gastroenterology and Hepatology, AZ Sint-Jozef Turnhout, Turnhout, Belgium; 4 12521 Gastroenterology and Hepatology, Second Military Medical University, Shanghai, China; 5 Endoscopy Center, Shanghai Eastern Hepatobiliary Hospital, Shanghai, China; 6 159841 Department of Surgery, Helsinki University Central Hospital, Helsinki, Finland; 7 9820 Department of Gastroenterology, Nottingham City Hospital NHS Trust, Nottingham, United Kingdom of Great Britain and Northern Ireland; 8 NIHR Nottingham Digestive Diseases Biomedical Research Unit, Nottingham University Hospitals NHS Trust and the University of Nottingham, Nottingham, United Kingdom of Great Britain and Northern Ireland

**Keywords:** Pancreatobiliary (ERCP/PTCD), Pancreatobiliary (ERCP/PTCD), ERC topics, Quality and logistical aspects, Training

## Abstract

**Background and study aim** Simulator-based training has been extensively studied in training gastroduodenoscopy and colonoscopy and shown to significantly improve learning curves of novices. Data on simulator-based training in endoscopic retrograde cholangiopancreatography (ERCP) are scarce. We aimed to determine the impact of 2-day intensive hands-on simulator training on the course of the learning curve of novice trainees.

**Methods** We conducted a prospective cohort study using a validated mechanical ERCP simulator (Boškoski-Costamagna ERCP Trainer). Six trainees were allocated to the simulation course program (SG). Each of these trainees were paired with an endoscopy trainee starting regular ERCP training at the same center who had no exposure to a simulation course program (control group; CG). The course included lectures, live ERCP demonstrations, and hands-on ERCP training to educate trainees in basic techniques related to cannulation, stent placement, stone extraction and stricture management. After the course, both the SG and CG started formal ERCP training in their respective centers. The Rotterdam Assessment Form for ERCP was used to register each performed ERCP. Simple moving average was applied to create learning curves based on successful common bile duct (CBD) cannulation. Outcomes were plotted against a historical cohort (HC).

**Results** Thirteen trainees were included, six trainees in the SG and seven trainees in the CG, with a total of 717 ERCPs. Mean successful ERCP cannulation rate was higher for the simulator group at baseline compared to both CG and HC, 64% versus 43% and 42%, respectively. Differences became less explicit after 40 ERCPs, but persisted until a median of 75 ERCPs.

**Conclusions** We demonstrate that 2-day hands-on simulator-based ERCP training course has a positive effect on the learning curves of ERCP trainees and should be considered an integral part of the training curricula for ERCP to develop skills prior to patient-based training.

## Introduction

Endoscopic retrograde cholangiopancreatography (ERCP) is a technically challenging procedure with significantly higher complication rates compared to standard endoscopic procedures 1 2 . The outcome of ERCP is highly operator-dependent. Complications are more likely to occur when an ERCP is performed by an inexperienced endoscopist 3 . Extensive training and procedural exposure is required to gain both technical and cognitive competency in ERCP.

To date, novice ERCP-ists are trained in a clinical setting through supervised, hands-on training in real patients. Advantages of the current training system include, among others, the opportunity to gain immediate feedback by an experienced endoscopist. However, this approach does have distinct disadvantages. This type of training is an example of learning by “trial and error” and potentially increases the risk of complications and patient discomfort. Additionally, it adds time and costs to each procedure affecting total capacity and financial resources 4 . Trainees operate in a stressful environment, which may be less suited to process feedback appropriately with the risk of being exposed to an overload of new information. The optimal methodology to acquire competence in ERCP is an ongoing topic of debate. Historically, it was assumed that competence is gained when a minimum number of ERCP procedures is performed, with guidelines recommending threshold numbers, varying from 100 to 200 ERCP procedures, at which time a trainee should reach an 80% common bile duct (CBD) cannulation success rate 5 . In a study by Verma et al *.* 6 , however, it was shown that a CBD cannulation rate of more than 80% was achieved only after 400 supervised procedures. As a result of this study there has been a shift to a more individualized approach, considering that individual trainees develop endoscopic skills at a different pace 7 . The specific role of simulators in training ERCP have not been defined yet. The outcome of simulator-based training on competence in gastroduodenoscopy and colonoscopy have been extensively studied, demonstrating that novices gain significant experience by training on simulators before they are exposed to real patients 8 9 . The improvement in performance seems most prominent in the early phase of the training. For example, a study by Koch et al *.* evaluating simulator training in colonoscopy demonstrated that there was no further improvement after 60 procedures 10 . Data on simulator training for training ERCP are scarce. Previously, our study group validated a novel mechanical ERCP trainer, the Boškoski-Costamagna ERCP trainer 11 12 .

For this study, our primary aim was to assess whether a 2-day intensive hands-on training including the use of the Boškoski-Costamagna ERCP Trainer in novice ERCP trainees at the start of patient-based training resulted in an acceleration and improvement of their learning curve. Our secondary aim was to establish to what extent this advantage would last.

## Materials and methods

### Study design

This was a prospective cohort study conducted in seven tertiary referral centers in five countries ( **Supplementary Table 1** ) **.** A total of 13 endoscopy trainees participated in this study. Allocation of participants was not strictly random, but was based on registration for a 2-day ERCP simulator training course in Rome, Italy. Participation in the course was allowed for endoscopists at the beginning of their ERCP careers. The simulator course participants (SG, simulator group) were paired with a starting advanced endoscopy trainee at their respective institution to form a control group (CG, control group). At study onset, all subjects completed a questionnaire to determine their demographics, baseline endoscopic experience, ERCP-specific experience, and simulator familiarity.

### Simulator

**Fig. 1:**
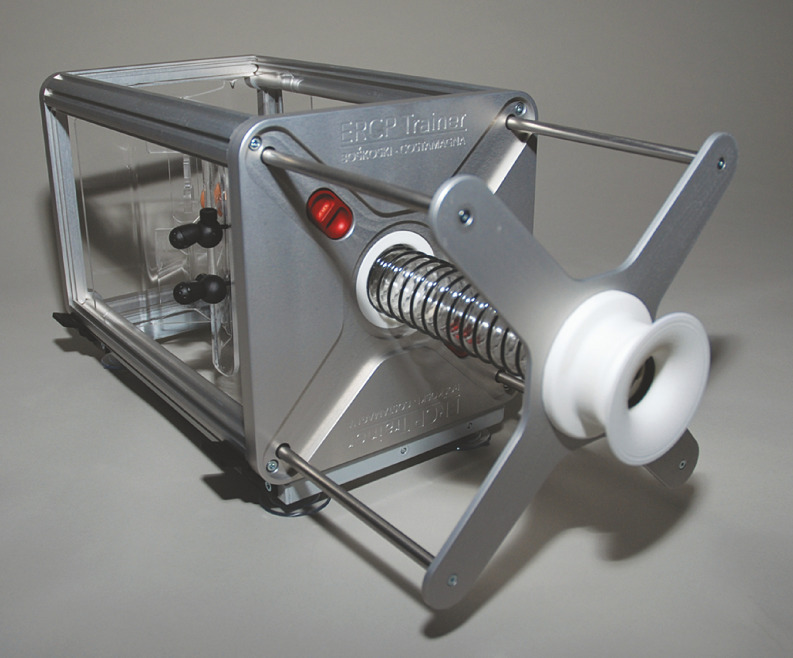
The Boškoski-Costamagna ERCP Trainer.

The second-generation Boškoski-Costamagna ERCP Trainer (Cook Medical, Limerick, Ireland) was used in this study. This is a mechanical simulator and consists of a metal framework with the esophagus, stomach, and duodenum constructed from plastic. The simulator has been designed to train novice endoscopists on correct positioning of the endoscope, assuming that a successful ERCP is largely dependent upon the ability to achieve an optimal position of the endoscope in front of the papilla. The simulator enables use of a real duodenoscope and commercially available accessories. Training options include positioning of the endoscope in front of the papilla, cannulation of the CBD, cannulation of the pancreatic duct, removal of a CBD stone using a coffee bean, and stent placement. In this model, it was not yet possible to practice sphincterotomy. A small video camera provides simulated fluoroscopy. The simulator has been previously described in detail in a validation study 11 . The simulator is depicted in Fig. 1 **.**

### Two-day ERCP training program

The 2-day ERCP simulator training course is hosted in the European Endoscopy Training Centre (EETC) at the Gemelli University Hospital, Rome, Italy, and a comparable training setting has been set up at the Eastern Hepatobiliary Hospital, Second Military Medical University, Shanghai, China. The course includes lectures, live ERCP demonstrations, and hands-on ERCP training to teach trainees the basic techniques related to cannulation, stent placement, stone extraction, and stricture management. The program starts with a lecture on the basics of cannulation and sphincterotomy techniques, followed by a 2-hour session of live demonstrations focusing on the position of the endoscope and cannulation techniques. In the afternoon, trainees receive hands-on training on the simulator for at least 3.5 hours. The second day starts with a lecture on prevention of biliopancreatic complications followed by live demonstration with additional lectures on stent and stricture management.

Subsequently, the trainees are again exposed to hands-on training for at least 2.5 hours. During these hands-on training sessions, trainees are able to extensively practice the various techniques under the supervision of experienced endoscopists. The course content was delivered by the EETC faculty and the visiting faculty. The group comprised a maximum of 10 trainees and at least one or two ERCP practitioners of the visiting faculty were present. Five Boškoski-Costamagna ERCP Trainers were available for hands-on training. Two trainees were allocated per simulator. Both trainees alternated in their role as assistant and endoscopist. ERCP training was performed using a standard therapeutic duodenoscope (PENTAX Medical, Hoya Corp., Tokyo, Japan) and commercially available accessories from Cook Medical, Limerick, Ireland.

### Rotterdam Assessment Form for ERCP

Both the SG and CG started their formal ERCP training in a real-life setting in patients at their own departments. The Rotterdam Assessment Form for ERCP (RAF-E) was used to register and score each performed ERCP. In 2014, Ekkelenkamp et al. 13 demonstrated that this self-assessment tool allows both trainees and trainers to gain insight in procedural quality of ERCP procedures by means of proposed ERCP quality indicators 14 . The tool was used in a second study to evaluate the learning curves of novice trainees 15 . The RAF-E form is largely based on previously validated assessment tools. All ERCPs performed in this study were part of routine clinical care performed at the participating centers, regardless of the indication for ERCP and a previously performed sphincterotomy. Participants completed a RAF-E form after each procedure.

### Historical cohort

Results in terms of successful biliary cannulation rates for both SG and CG were plotted against a historical cohort (HC) of 15 ERCP trainees. In 2014, Ekkelenkamp et al. *,* from the same research group, published the results of a prospective study evaluating the ERCP learning curves of 15 novice trainees in the Netherlands 15 . A total of 1541 ERCPs were included in the study. The trainees followed their regular training program, without previous ERCP simulator training, and documented each performed ERCP using the RAF-E.

### Outcome measures

The main outcome measure was successful CBD cannulation rate. In a previous study by our group 15 , we have demonstrated that CBD cannulation can be regarded as a surrogate marker for procedural competence. The curve for CBD cannulation is comparable to those for therapeutic interventions such as stent placement and sphincterotomy. This means that the learning curve for overall CBD cannulation success can be used for quick information about whether an individual trainee is progressing according to the expected group learning curve or not. It reflects the corresponding learning curves for therapeutic interventions. For this reason, our focus in the current study was solely on cannulation success rates. Therapeutic interventions and procedural success were not necessarily reported for all procedures.

### Statistical analysis

Statistical analyses were performed using SPSS 25.0 software (IBM Corp: Armonk, New York, United States). Baseline characteristics, group averages and standard deviations were presented in mean, median with standard deviation or interquartile range, respectively. A two-sided *P* <0.05 was considered significant. Graphs were created with standard software.

A simple moving average technique was used to analyze the ERCP learning curves of the trainees based on successful cannulation rates. The moving average technique depicts data points by creating a series of averages of different subsets of the complete data set. A moving average creates a trend line that partially compensated for outlying results and displays a learning curve over time that is easier to interpret compared to a loose set of data points. It is a method that is used in a number of studies regarding learning curves 15 16 17 . The mean number of successful cannulations of the CBD was calculated for each trainee over blocks of 10 ERCP procedures. A rising moving average indicates a positive learning curve plotted in successful CBD cannulation rates.

## Results

A total of 13 trainees (9 male) from six countries were included in this study. The SG consisted of six trainees. The remaining seven trainees were assigned to the CG. The mean age of the trainees was 32 years. Five trainees (38.5%) had been trained previously on a simulator (gastroduodenoscopy or colonoscopy simulator training), two were assigned to the SG and three trainees to the CG. Ten trainees had no previous ERCP experience, two trainees had a maximum of 10 previously performed ERCPs (in each group one trainee), and one trainee had performed a maximum of 20 procedures and was included in CG. The SG performed around 30 procedures per person during the 2-day training course. Overall, the group of trainees performed a total of 717 ERCPs at their own institutions. The median number of ERCP procedures per trainee performed during the study period was 24 procedures with a broad range of nine to 153 procedures. The median number of ERCPs in the simulator training group was significantly higher than in the conventional training group (56 versus 22 procedures, *P* = 0.002). The overall percentage of ERCPs performed in patients with a native major papilla was 52.4% and did not differ significantly between groups. A statistically significant difference between groups was seen in ERCP difficulty degree ( *P* = 0.001), with more difficult ERCPs in the SG. Baseline characteristics are outlined in Table 1 **.**

**Table:** **Table 1** Baseline characteristics.

	Simulator group	Conventional group	Total	P
Trainees	6	7	13	
Male, n (%)	5 (83.3)	4 (57.1)	9 (69.2)	0.190
Age in years, mean (SD)	33.0 (1.0)	31.2 (2.5)	32.0 (2.1)	
Simulator familiarity (%)	2 (33.3)	3 (42.9)	5 (38.5)	0.436
Patient-based ERCP procedures performed, n (%)	383 (53.3)	334 (46.5)	717	0.002
Median number of ERCP procedures, n (range)	56 (13–140)	22 (9–153)	24 (9–153)	
Indication				0.089
Reaching and papilla cannulation, n (%)	16 (4.2)	20 (6.0)	36 (5.0)	
Complete stone extraction CBD, n (%)	120 (31.3)	99 (29.6)	219 (30.5)	
Endoprothesis – stenosis CBD, n (%)	121 (31.6)	91 (27.2)	212 (29.6)	
Metal stent – stenosis CBD, n (%)	73 (19.1)	64 (19.2)	137 (19.1)	
Endoprothesis bile leakage, n (%)	13 (3.4)	6 (1.8)	19 (2.6)	
Therapy chronic pancreatitis, n (%)	7 (1.8)	17 (5.1)	24 (3.3)	
Other, n (%)	33 8.6)	37 (11.1)	70 (9.8)	
Difficulty degree, n (%)				0.001
1	187 (48.8)	211 (63.2)	398 (55.5)	
2	161 (42.0)	102 (30.5)	263 (36.7)	
3	35 (9.1)	21 (6.3)	56 (7.8)	
Native papillary anatomy, n (%)				0.067
Yes	211 (55.1)	165 (49.4)	376 (52.4)	
No	172 (44.9)	169 (50.6)	341 (47.6)	
Previous ERCP failure, n (%)				0.336
Yes	41 (10.7)	42 (12.6)	83 (11.6)	
No	262 (68.4)	211 (63.2)	473 (66.0)	
Not applicable	80 (20.9)	81 (24.3)	161 (22.5)	
ASA Score, n (%)				0.000
ASA 1	58 (15.8)	60 (21.6)	118 (18.3)	
ASA 2	198 (54.1)	176 (63.3)	374 (58.1)	
ASA 3	99 (27.0)	38 (13.7)	137 (21.3)	
ASA 4	8 (2.2)	4 (1.4)	12 (1.9)	
ASA 5	3 (0.8)	0 (0)	3 (0.5)	
CBD, common bile duct; ERCP, endoscopic retrograde cholangiopancreatography; ASA, American Society of Anesthesiologists.				

### Moving average curve

**Fig. 2:**
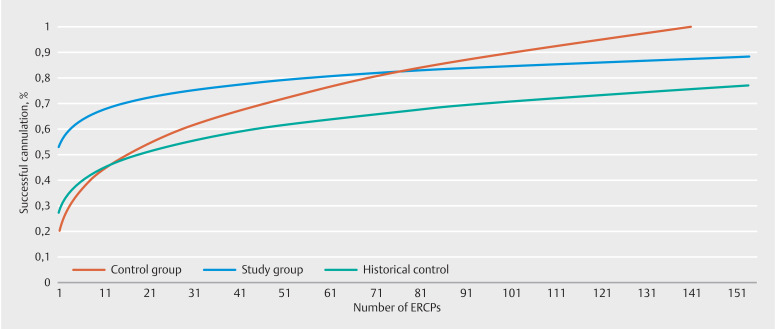
Moving average curve for successful CBD cannulation in patient-based ERCP.

The simple moving average of SG versus CG and HC is plotted in Fig. 2 . The X axis signifies the cumulative ERCP procedure number and the Y axis represents the percentage of successful CBD cannulation in patient-based ERCP. Mean successful ERCP cannulation rate was higher for the simulator group at baseline (moving average after the first 10 ERCPs) compared to both CG and HC, 64% versus 43% and 42%, respectively. After 40 ERCPs, the differences in successful CBD cannulation become less explicit between the SG and both the CG and HC, but persisted until a median of 75 ERCPs. At this point. a successful CBD cannulation rate of 82% is seen in both the SG and CG. From this point on the available data were too limited to detect a statistical difference between the learning curves. The HC did not cross the line of the SG and shows a successful cannulation rate of 68% after 75 procedures.

## Discussion

In this prospective study, we demonstrated that novice ERCP trainees gain significant experience by training on the mechanical ERCP simulator before they are exposed to real patients. The 2-day hands-on training course had a positive effect on the performance of trainees compared to the CG. The effect of simulation-based ERCP training on patient-based performance lasted up to around 75 ERCPs.

Despite growing awareness that procedure numbers are an inadequate means to define competence in ERCP, it is still the predominant methodology used to define the competence of trainees in most training curricula. Several studies have demonstrated that trainees reach competency at various points in training and that training guidelines underestimate the number of ERCPs necessary to achieve competence 15 18 . A recent published review by Voiosu et al. 19 provides an overview of the current studies concerning trainee competence in ERCP. Importantly, most trainees do not reach predefined competence thresholds, supporting the idea that a more individualized approach is necessary. The role of simulator-based training in ERCP has not been defined yet, but the essence of simulation-based training is to provide trainees with opportunities to understand the anatomy and to become familiar with both the endoscope and accessories at their own pace without compromising patient safety. Simulation-based training creates a unique and safe learning environment in which to teach trainees the basic skills of ERCP and to provide the trainer with insights into the learning curve of trainees with the opportunity for timely intervention.

According to our study results, compared to on-the-job learning, a 2-day hands-on course in a stress-free simulated training environment has a positive impact on the subsequent learning curve when performing real-life ERCP procedures with a beneficial effect that lasts up to around 75 procedures. The effect was demonstrated using successful CBD cannulation rate as the outcome measure. CBD cannulation can be regarded as a surrogate marker for procedural competence, as seen in a previous study by Ekkelenkamp et al 15 .

The effect of simulator-based training is observed immediately from the beginning of patient-based ERCP performance when measuring successful cannulation in the first 10 procedures with a successful cannulation rate of 64% in the SG compared to 43% in the CG. Compared to the HC, the CG demonstrates a steeper learning curve potentially indicating that training options have improved over the last years. Our data correspond with previous simulator training studies in endoscopy, mostly in the field of training colonoscopy, demonstrating a significant benefit of simulator training in the early learning curve 9 .

Limited data available for simulator training in ERCP concern mainly the ERCP mechanical simulator (EMS Trainer) demonstrating that trainees who underwent simulator-based training achieved higher success rates with selective and deep cannulation of the CBD compared to the CG in the first months of training 20 21 22 . A potentially valuable addition to the Boškoski-Costamagna ERCP Trainer is the synthetic papilla, which can be used to train sphincterotomy using commercially available sphincterotomes and needle knives. The papilla has been validated in a previous study by our study group 12 , but was not yet available for training during the study period.

Some limitations of our study need to be considered when interpreting our results. The number of participating trainees was limited. Results are representative for the trainees with no or very little real-life ERCP exposure. It cannot be inferred how these results translate to trainees with limited but more extensive experience (e.g. 50–100 procedures). Dropout of participants who did not continue ERCP training, either due to insufficient training resources at their respective facilities or the fact that there was a shift in priorities during their specialty training, prohibit drawing conclusions beyond 75 procedures. This was not a formal randomized controlled trial but a paired controlled cohort study with inclusion of the participants of the intervention group based on their specific interest in attending an ERCP training course, which may have introduced selection bias. Although the 2-day ERCP simulator training course was structured and equal for all participants, the real-life training at their respective institutions was not and was left to the discretion of the local team. To attempt to partially overcome potential bias in this regard, a trainee from the same training center was included.

We have included all the performed ERCPs by the trainees regardless of indication or papilla status. By excluding all cases with previous sphincterotomy, potential bias might be introduced because these cases do add to the learning curve and might not be equally distributed chronologically during the training period. By including all cases regardless of indication or papilla status, we tried to minimize bias in that regard.

Another fact that needs to be taken into account is that the SG performed more ERCPs and also more complex ERCPs in comparison to the CG. Although allocation to the SG or CG group was done after participants had expressed their desire to receive ERCP training, we cannot rule out potential bias that the most motivated participants entered the simulator course. Another explanation might also be that after a successful simulator course, participants experienced a shorter learning curve and were more prepared to overcome some of the difficulties that ERCP brings.

This study, however, provides ample rationale that simulator training for early-learning-phase trainees has a beneficial effect and should be considered to have a formal role in ERCP training curricula. Simulator training provides trainees with the opportunity to perform the procedure multiple times without risks at their own pace before performing the procedure on a real patient. It may be inferred that apart from potentially decreasing complication risks and patient discomfort, less time may be spent per patient in the early phase of training, thereby increasing procedural capacity. It is our belief that based on our results, further research is warranted to determine the optimal duration and extent of simulator training, the optimal simulator to be used, and finally, how such training should be implemented in the training curricula.

## Conclusions

In conclusion, we demonstrate a positive effect of simulator-based training during a 2-day hands-on training course in the early learning curve of ERCP trainees prior to patient-based training. Simulator training should be considered an integral part of the training curricula for ERCP.

## Supporting information

Supplementary material Zusatzmaterial

## References

[1] Andriulli A Loperfido S Napolitano G Incidence rates of post-ERCP complications: a systematic survey of prospective studies Am J Gastroenterol 2007 102 1781 1788 10.1111/j.1572-0241.2007.01279.x 17509029

[2] Cotton PB Endoscopic retrograde cholangiopancreatography: maximizing benefits and minimizing risks Gastrointest Endosc Clin N Am 2012 22 587 599 10.1016/j.giec.2012.05.002 22748250

[3] Jowell PS Baillie J Branch MS Quantitative assessment of procedural competence. A prospective study of training in endoscopic retrograde cholangiopancreatography Ann Intern Med 1996 125 983 989 10.7326/0003-4819-125-12-199612150-00009 8967710

[4] Baron TH Petersen BT Mergener K Quality indicators for endoscopic retrograde cholangiopancreatography Am J Gastroenterol 2006 101 892 897 10.1016/j.gie.2006.02.019 16635233

[5] ASGE Training Committee Jorgensen J Kubiliun N Endoscopic retrograde cholangiopancreatography (ERCP): core curriculum Gastrointest Endosc 2016 83 279 289 26708081 10.1016/j.gie.2015.11.006

[6] Verma D Gostout CJ Petersen BT Establishing a true assessment of endoscopic competence in ERCP during training and beyond: a single-operator learning curve for deep biliary cannulation in patients with native papillary anatomy Gastrointest Endosc 2007 65 394 400 17321237 10.1016/j.gie.2006.03.933

[7] Wani S Hall M Wang AY Variation in learning curves and competence for ERCP among advanced endoscopy trainees by using cumulative sum analysis Gastrointest Endosc 2016 83 711 719 e711 26515957 10.1016/j.gie.2015.10.022

[8] Ekkelenkamp VE Koch AD de Man RA Training and competence assessment in GI endoscopy: a systematic review Gut 2016 65 607 615 10.1136/gutjnl-2014-307173 25636697

[9] Singh S Sedlack RE Cook DA Effects of simulation-based training in gastrointestinal endoscopy: a systematic review and meta-analysis Clin Gastroenterol Hepatol 2014 12 1611 1623 e1614 10.1016/j.cgh.2014.01.037 24509241

[10] Koch AD Ekkelenkamp VE Haringsma J Simulated colonoscopy training leads to improved performance during patient-based assessment Gastrointest Endosc 2015 81 630 636 25475901 10.1016/j.gie.2014.09.014

[11] van der Wiel SE Koch AD Bruno MJ Face and construct validity of a novel mechanical ERCP simulator Endosc Int Open 2018 6 E758 E765 10.1055/s-0044-101754 29881768 PMC5989785

[12] van der Wiel SE Koch AD Bruno MJ Face validity of a synthetic papilla designed for biliary sphincterotomy training Endosc Int Open 2019 7 E757 E761 10.1055/a-0842-6369 31157293 PMC6524995

[13] Ekkelenkamp VE Koch AD Haringsma J Quality evaluation through self-assessment: a novel method to gain insight into ERCP performance Frontline Gastroenterol 2014 5 10 16 24416502 10.1136/flgastro-2013-100334 PMC3880906

[14] Baron TH Petersen BT Mergener K Quality indicators for endoscopic retrograde cholangiopancreatography Gastrointest Endosc 2006 63 S29 S34 10.1016/j.gie.2006.02.019 16564909

[15] Ekkelenkamp VE Koch AD Rauws EA Competence development in ERCP: the learning curve of novice trainees Endoscopy 2014 46 949 955 10.1055/s-0034-1377930 25208031

[16] Koch AD Haringsma J Schoon EJ Competence measurement during colonoscopy training: the use of self-assessment of performance measures Am J Gastroenterol 2012 107 971 975 22764019 10.1038/ajg.2011.481

[17] Siau K Crossley J Dunckley P Colonoscopy direct observation of procedural skills assessment tool for evaluating competency development during training Am J Gastroenterol 2020 115 234 243 31738285 10.14309/ajg.0000000000000426

[18] Wani S Keswani R Hall M A prospective multicenter study evaluating learning curves and competence in endoscopic ultrasound and endoscopic retrograde cholangiopancreatography among advanced endoscopy trainees: the Rapid Assessment of Trainee Endoscopy Skills Study Clin Gastroenterol Hepatol 2017 15 1758 1767 e1711 28625816 10.1016/j.cgh.2017.06.012 PMC7042954

[19] Voiosu T Balanescu P Voiosu A Measuring trainee competence in performing endoscopic retrograde cholangiopancreatography: A systematic review of the literature United European Gastroenterol J 2019 7 239 249 10.1177/2050640618817110 PMC6498806 31080609

[20] Lim BS Leung JW Lee J Effect of ERCP mechanical simulator (EMS) practice on trainees' ERCP performance in the early learning period: US multicenter randomized controlled trial Am J Gastroenterol 2011 106 300 306 10.1038/ajg.2010.411 20978485

[21] Leung JW Liao W-C Wang H-P A RCT of mechanical simulator practice and usual training vs. usual training on novice trainee clinical ERCP performance Gastrointest Endosc 2009 69 10.1016/j.gie.2009.03.204

[22] Meng W Yue P Leung JW Impact of mechanical simulator practice on clinical ERCP performance by novice surgical trainees: a randomized controlled trial Endoscopy 2020 52 1004 1013 10.1055/a-1217-6727 32869230

