# Supplementary material for: Impact of ERCP simulator training on early ERCP learning curves of novice trainees: a cohort study
Source: Endosc Int Open. 2023 Aug 1;11(8):E690–6. doi: 10.1055/a-2114-2842 (PMC10411077; doi:10.1055/a-2114-2842)
Supplement: Supplementary file 1 — Supplementary material [file 10-1055-a-2114-2842_21178307.pdf]

Supplementary material

SUPPLEMENTARY

Table 1: *List of participating advanced endoscopy training centers*

Erasmus University Medical Center	Rotterdam, The Netherlands
Academic Medical Center	Amsterdam, The Netherlands
Helsinki University Hospital	Helsinki, Finland
Turku University Hospital	Turku, Finland
Nottingham University Hospitals	Nottingham, United Kingdom
Eastern Hepatobiliary Surgery Hospital	Shanghai, China
Leuven University Hospital	Leuven, Belgium
